# Dendrimer-2PMPA selectively blocks upregulated microglial GCPII activity and improves cognition in a mouse model of multiple sclerosis

**DOI:** 10.7150/ntno.63158

**Published:** 2022-01-01

**Authors:** Kristen R. Hollinger, Anjali Sharma, Carolyn Tallon, Lyndah Lovell, Ajit G. Thomas, Xiaolei Zhu, Robyn Wiseman, Ying Wu, Siva P. Kambhampati, Kevin Liaw, Rishi Sharma, Camilo Rojas, Rana Rais, Sujatha Kannan, Rangaramanujam M. Kannan, Barbara S. Slusher

**Affiliations:** 1 Johns Hopkins Drug Discovery, Johns Hopkins University, Baltimore, MD, USA.; 2 Center for Nanomedicine, Department of Ophthalmology, Johns Hopkins University, Baltimore, MD, USA.; 3 Department of Neurology, Johns Hopkins University, Baltimore, MD, USA.; 4 Department of Psychiatry and Behavioral Sciences, Johns Hopkins University, Baltimore, MD, USA.; 5 Department of Pharmacology and Molecular Sciences, Johns Hopkins University, Baltimore, MD, USA.; 6 Department of Chemical and Biomolecular Engineering, Johns Hopkins University, Baltimore, MD, USA.; 7 Department of Anesthesiology and Critical Care Medicine, Johns Hopkins University, Baltimore, MD, USA.; 8 Kennedy Krieger Institute, Johns Hopkins University, Baltimore, MD, USA.; 9 Department of Neuroscience, Johns Hopkins University, Baltimore, MD, USA.; 10 Department of Medicine, Johns Hopkins University, Baltimore, MD, USA.; 11 Department of Oncology, Johns Hopkins University, Baltimore, MD, USA.

**Keywords:** multiple sclerosis, cognitive impairment, dendrimer, GCPII, NAAG

## Abstract

Cognitive impairment is a common aspect of multiple sclerosis (MS) for which there are no treatments. Reduced brain *N*-acetylaspartylglutamate (NAAG) levels are linked to impaired cognition in various neurological diseases, including MS. NAAG levels are regulated by glutamate carboxypeptidase II (GCPII), which hydrolyzes the neuropeptide to *N*-acetyl-aspartate and glutamate. GCPII activity is upregulated multifold in microglia following neuroinflammation. Although several GCPII inhibitors, such as 2-PMPA, elevate brain NAAG levels and restore cognitive function in preclinical studies when given at high systemic doses or via direct brain injection, none are clinically available due to poor bioavailability and limited brain penetration. Hydroxyl-dendrimers have been successfully used to selectively deliver drugs to activated glia.

**Methods:** We attached 2-PMPA to hydroxyl polyamidoamine (PAMAM) dendrimers (D-2PMPA) using a click chemistry approach. Cy5-labelled-D-2PMPA was used to visualize selective glial uptake *in vitro* and *in vivo*. D-2PMPA was evaluated for anti-inflammatory effects in LPS-treated glial cultures. In experimental autoimmune encephalomyelitis (EAE)-immunized mice, D-2PMPA was dosed biweekly starting at disease onset and cognition was assessed using the Barnes maze, and GCPII activity was measured in CD11b+ hippocampal cells.

**Results:** D-2PMPA showed preferential uptake into microglia and robust anti-inflammatory activity, including elevations in NAAG, TGFβ, and mGluR3 in glial cultures. D-2PMPA significantly improved cognition in EAE mice, even though physical severity was unaffected. GCPII activity increased >20-fold in CD11b+ cells from EAE mice, which was significantly mitigated by D-2PMPA treatment.

**Conclusions:** Hydroxyl dendrimers facilitate targeted drug delivery to activated microglia. These data support further development of D-2PMPA to attenuate elevated microglial GCPII activity and treat cognitive impairment in MS.

## Introduction

Multiple sclerosis (MS) is an autoimmune disease of the central nervous system (CNS). There are over 15 disease-modifying therapies approved by the FDA to treat the various subtypes of MS, but none target cognitive impairment that affects over half of all MS patients. This unmet treatment need is critical to explore, as MS-related learning and memory impairments have a measurable negative impact on quality of life, cost of living, and employability 1-4.

The enzyme glutamate carboxypeptidase II (GCPII) metabolizes the neuropeptide *N*-acetylaspartyl glutamate (NAAG) into *N*-acetyl aspartate (NAA) and glutamate 5. NAAG is one of the most abundant neuropeptides in the mammalian brain 6 and regulates neural activity through its agonist activity at the metabotropic glutamate receptor 3 (mGluR3) 7. Previous studies have demonstrated a significant correlation between brain NAAG levels and cognition in MS patients, with higher NAAG levels associated with better cognitive function 8. Furthermore, in an animal model of MS, the potent (IC_50_=300 pM) and selective GCPII inhibitor 2-(phosphonomethyl)-pentanedioic acid (2-PMPA) was shown to enhance brain NAAG levels and improve cognitive function 8, 9. Despite this strong therapeutic data, existing GCPII inhibitors have poor bioavailability and brain penetration and therefore are unsuitable for clinical translation to MS patients.

Nanotechnology has shown promise in the development of targeted brain delivery systems for therapeutics with poor pharmacokinetic profiles 10, with some advancing to clinical trials 11. Among the nanoparticles for biological applications, hydroxyl dendrimers have shown promise as targeted intracellular delivery systems, due to their size (~4-10 nm) and surface attributes 12. Dendrimers are monodispersed and multivalent macromolecules with tailorable surface functionalities upon which bioactive molecules such as drugs, targeting ligands and/or imaging dyes can be covalently conjugated. Previous studies have demonstrated that systemically administered hydroxyl polyamidoamine (PAMAM) dendrimer-drug conjugates do not cross the blood brain barrier (BBB) and are rapidly cleared under normal conditions, but under neuroinflammatory conditions can cross the impaired BBB and diffuse in the brain parenchyma where they are taken up by activated and phagocytic glia 13-17. Moreover, the dendrimer-drug conjugates are consistently multifold more potent and efficacious when directly compared to the equivalent amount of the free drug, likely due to their targeted delivery 18, 19. Targeted hydroxyl PAMAM dendrimer brain delivery has been demonstrated in multiple animal models of neuroinflammation, including cerebral palsy, inflammatory preterm birth injury, and Rett syndrome 13, 14, 20-23. We therefore hypothesized that conjugating 2-PMPA to a hydroxyl dendrimer would enable its preferential delivery to activated microglia in EAE-immunized mice. This targeting is relevant as both GCPII and NAAG are known to be measurably upregulated in activated microglia 24, 25, making dendrimers a potentially efficacious GCPII inhibitor delivery vehicle for the treatment of cognitive impairment in MS.

Herein, we conjugated 2-PMPA to the surface of hydroxyl PAMAM dendrimers (D-2PMPA) and evaluated its glial uptake, anti-inflammatory efficacy in glial cultures, and behavioral effects and target engagement in EAE-immunized mice.

## Material and methods

### Synthesis of D-2PMPA and Cy5-D-2PMPA conjugates

Reagents and solvents were purchased from Sigma Aldrich or Fisher Scientific and were used as received unless otherwise stated. 2-PMPA (**compound 1**) was purchased from Sigma Aldrich (St. Louis, MO). Ethylenediamine-core PAMAM-OH dendrimer generation 4 having 64 hydroxyl end-groups, Pharma grade (**compound 3, D-OH**) was received as a methanolic solution from Dendritech. Before use, the methanol was evaporated and dendrimer was further purified to remove generational impurities by dialysis. The dendrimer was solubilized in water and dialyzed against water using dialysis membrane of 3kDa. The dialysis membranes were purchased from Spectrum Laboratories Inc. Azido-PEG-11-alcohol was purchased from Broad Pharm and Cy5 NHS ester was purchased from GE healthcare and used as received. Deuterated solvents for NMR spectroscopy such as (DMSO‑*d6*), methanol (CD_3_OD), water (D_2_O) and chloroform (CDCl_3_) were purchased from Sigma. D-Cy5 was synthesized using our previously published protocol 23, 26.

### Synthesis of compound 2

To a stirring solution of 2-PMPA (500 mg, 2.211 mmoles) in anhydrous DMF (5 mL), DMAP (300 mg, 2.455 mmoles) and EDC (577, 3.00 mmoles) were added. The reaction mixture was stirred for 10 minutes. This was followed by the addition of azido-PEG-11-alcohol (1.06 g, 2.010 mmoles). The solution was stirred at room temperature overnight. Upon completion, the DMF was evaporated and the crude product was purified using reverse-phase column purification using acetonitrile and water. The pure fractions were evaporated to afford compound **2** as viscous liquid. Yield: 41%

**^1^H NMR** (500 MHz, D_2_O) δ 4.27 (t, 2H), 3.78 (t, 2H), 3.70 (s, 40H), 3.50 (t, 2H), 2.79-2.69 (m, 1H), 2.50 (t, 2H), 2.18-2.07 (m, 1H), 2.08-1.92 (qd, 2H), 1.92-1.79 (m, 1H) (**Figure S1**).

HRMS: m/z: calculated: 735.72, found: 758.31 [M+Na]^+^ (**Figure S2**).

### Synthesis of compound 4

To a stirring solution of D-OH (**3**, 2.00 g, 0.140 mmoles) in anhydrous DMF, 5-hexynoic acid (235 mg, 2.10 mmoles) followed by the DMAP (256 mg, 2.10 mmoles) and EDC (537mg, 2.801 mmoles) were added. The stirring was continued for 24 hours at room temperature. The reaction mixture was then diluted with DMF and dialyzed against DMF for 6 hours followed by the overnight water dialysis. The solvents were changed frequently during dialysis. The aqueous solution was then lyophilized to obtain compound** 4** as white solid. Yield: 91%

**^1^H NMR** (500 MHz, D_2_O) δ 7.93 (m, D-internal amide *H*), 4.72 (bs, D-O*H*), 4.01 (t, ester -C*H*_2_), 3.49-3.21 (m, D--C*H*_2_), 3.11 (m, D and linker-C*H*_2_), 2.79-2.58 (m, D and linker-C*H*_2_), 2.49-2.30 (m, D-C*H*_2_), 2.31-2.05 (m, D-C*H*_2_), 1.69 (t, linker-C*H*_2_) (**Figure S3**).

### Synthesis of compound 5

To a stirring solution of compound **4** (1.9 g, 0.123mmoles) and Compound **2** (1.178 g, 1.604 mmoles) in DMF:THF (1:1), CuSO_4_.5H_2_O (16.844 mg, 0.067 mmoles) dissolved in 1mL water was added. This was followed by the addition of sodium ascorbate (26.804 mg, 0.135 mmoles) dissolved in 1 mL water. The reaction was carried out at 50 °C in a microwave reactor for 6 hours. Upon completion, the reaction mixture was diluted with water, EDTA solution (1 mL) was added and stirred for 1 hour. The solution was then transferred to a dialysis membrane (cut-off 1000 Da) and dialyzed against water for 15 hours. The aqueous solution was then lyophilized to afford the pure product as white solid. Yield: 82%

**^1^H NMR** (500 MHz, D_2_O) δ 7.87 (s, triazole *H*), 4.58 (t, linker -C*H*_2_), 4.15 (t, linker -C*H*_2_), 3.92 (t, ester -C*H*_2_), 3.83-3.52 (m, PEG H and dendrimer -C*H*_2_), 3.53-3.23 (m, D- and linker-C*H*_2_), 3.16-2.65 (m, D- and linker-C*H*_2_), 2.66-2.34 (m, D-C*H*_2_), 2.24-2.06 (m, PMPA *H*), 2.04-1.84 (m, PMPA *H*), 1.85-1.54 (m, PMPA *H*) (**Figure S4**).

**HPLC:** Retention time: 22.99 minutes, purity: 99.69% (**Figure S5**);

**MALDI:** Theoretical 22,589; Found: 21,881 Da (**Figure S6**).

### Synthesis of compound 6

To a stirring solution of compound **4** (810 mg, 0.053 mmoles) in DMF (10 mL), GABA-BOC-OH (53.74 mg, 0.265 mmoles) followed by DMAP (33 mg, 0.270 mmoles) and EDC (33 mg, 0.427 mmoles) were added. The reaction was stirred at room temperature for 24 hours. The solution was then transferred to a dialysis membrane (cut-off 1000 Da) and dialyzed against DMF followed by water for 15 hours. The aqueous solution was then lyophilized to afford the pure product as white solid. Yield: 86%

**^1^H NMR** (500 MHz, DMSO) δ 8.09-7.68 (m, D-internal amide *H*), 6.83 (s, N*H* BOC), 4.71 (bs, D-O*H*), 4.07-3.95 (m, ester -C*H*_2_ of both linkers), 3.49-3.21 (m, D- and linker-C*H*_2_), 3.21-2.99 (m, D-C*H*_2_), 2.99-2.83 (D-C*H*_2_), 2.83-2.56 (D- and linker-C*H*_2_), 2.47-2.33 (D-C*H*_2_), 2.31-1.98 (m, D- and linker-C*H*_2_), 1.59 (t, linker-C*H*_2_), 1.37 (s, BOC *H*) (**Figure S7**).

### Synthesis of compound 7

To a solution of compound **6** (200 mg) in DCM (7 mL), trifluoroacetic acid (3 mL) was added. The solution was vigorously stirred at room temp for 12 hours. DCM was then evaporated and TFA was removed by co-evaporation with methanol. This process was repeated several times. The trace solvents were removed using high vacuum to afford compound **7** as TFA salt in quantitative yield.

**^1^H NMR** (500 MHz, DMSO) δ 8.67-8.13 (m, D-internal amide *H*), 7.91 (s, -N*H*_2_), 4.07-3.95 (m, ester -C*H*_2_ of both linkers), 3.69-3.26 (m, D- and linker-C*H*_2_), 3.26-2.93 (m, D- and linker-C*H*_2_), 2.87-2.57 (m, D- and linker-C*H*_2_), 2.40 (m, D-C*H*_2_), 1.79 (t, linker-C*H*_2_) (**Figure S8**).

### Synthesis of compound 8

To a stirring solution of compound **7** (220 mg, 0.014 mmoles) in DMF, PMPA-PEG-azide (144.06 mg, 0.196 mmoles) followed by a catalytic amount of CuSO_4_.5H_2_O (1mg) in water (1 mL) was added. The reaction was stirred for 5 minutes. This was then followed by the addition of sodium ascorbate (2 mg) in water (1 mL). The reaction was placed in the microwave at 50 °C for 8 hours. Upon completion, the reaction mixture was diluted with water, and EDTA solution (1 mL) was added and stirred for 1 hour. The solution was then transferred to a dialysis membrane (cut-off 1000 Da) and dialyzed against water for 15 hours. The aqueous solution was then lyophilized to afford the pure product as white solid. Yield: 79%

**^1^H NMR** (500 MHz, DMSO) δ 8.30-7.74 (m, D-amide *H* and triazole *H*), 4.47 (t, linker -C*H*_2_), 4.06 (m, ester -C*H*_2_), 3.79 (t, linker -C*H*_2_), 3.58-3.28 (m, D- and linker-C*H*_2_), 3.24-2.98 (m, D- and linker-C*H*_2_), 2.93-2.59 (m, D- and linker-C*H*_2_), 2.40-1.98 (m, D-C*H*_2_), 1.96-1.38 (m, PMPA *H* and linker *H*) (**Figure S9**).

### Synthesis of compound 9

To a stirring solution of compound **8** (130 mg, 0.005 mmoles) in DMF (5 mL), DIPEA (0.1 mL) followed by Cy5-NHS ester (5.06 mg, 0.008 mmoles) dissolved in DMF (1 mL) was added. The reaction was stirred at room temperature for 24 hours. The solution was then transferred to a dialysis membrane (cut-off 1000 Da) and dialyzed against DMF followed by water for 10 hours. The aqueous solution was then lyophilized to afford the pure product as blue solid. Yield: 86%

**^1^H NMR** (500 MHz, DMSO) δ 8.41-8.70 (m, Cy5 *H*), 8.25-7.71 (m, D-amide *H* and triazole *H*), 7.77-7.60 (m, Cy5 *H*), 7.36-7.28 (m, Cy5 *H*), 6.62-6.51 (m, Cy5 *H*), 6.31-6.26 (m, Cy5 *H*), 4.47 (t, linker -C*H*_2_), 4.06 (m, ester -C*H*_2_), 3.79 (t, linker -C*H*_2_), 3.70-3.22 (m, D- and linker-C*H*_2_), 3.11-2.59 (m, D- and linker-C*H*_2_), 2.32-1.97 (m, D-C*H*_2_), 1.92-1.75 (m, PMPA *H* and linker *H*), 1.75-1.35 (m, PMPA *H*) (**Figure S10**).

**HPLC:** Purity 99.9%, Retention time: 22.5 minutes (**Figure S11**).

### Compound characterization

#### Nuclear Magnetic Resonance (NMR) spectroscopy

^1^H NMR spectra were logged at 500MHz using Bruker spectrometer at 25 °C. The chemical shifts of the residual protic solvent were reported in ppm relative to the internal trimethylsilane standard (δ = 0 ppm); CDCl_3_ (^1^H, δ = 7.27 ppm; ^13^C, δ = 77.0 ppm (central resonance of the triplet)), D_2_O (^1^H, δ = 4.79 ppm); and DMSO-*d*6 (^1^H, δ = 2.50 ppm) were used for chemical shifts calibration. The chemical shift multiplicities are abbreviated as follows: s = singlet, d = doublet, t = triplet, q = quartet, m = multiplet, and br = broad.

#### High Performance Liquid Chromatography (HPLC)

The purities of the intermediates and the final conjugates were analysed using HPLC (Waters Corporation, Milford, MA) equipped with a 2998 photodiode array detector, a 2475 multi λ fluorescence detector, a 1525 binary pump, and an in-line degasser AF. The HPLC was interfaced with Waters Empower software. A C18 symmetry 300, 5µm, 4.6x250mm column from Waters was used. The HPLC chromatograms were recorded at 210nm (dendrimer and 2-PMPA absorption), for D-2PMPA and at 650nm wavelength (Cy5 absorption) for Cy5-D-2PMPA. A gradient flow was used using a mobile phase consisting of buffer A: 0.1% TFA and 5% ACN in water and buffer B: 0.1% TFA in ACN). The gradient started from 100:0 (A:B) gradually increasing to 50:50 (A:B) at 20 min, finally returning to 100:0 (A:B) at 40 minutes maintaining a flow rate of 1 mL/min. For the purification of drug linker, semi-preparative HPLC from Shimadzu was used using same method with a flowrate of 5 mL/min.

#### Mass spectroscopy

High resolution mass spectrometry (HRMS) was performed on Bruker microTOF-II mass spectrometer using ESI in the positive mode and direct flow sample introduction in CH_3_CN/ H_2_O (9:1) solvent system. The empirical formula confirmation was obtained by protonated molecular ions [M + nH]^n+^ or adducts [M + nX]^n+^ (X = Na).

Matrix assisted laser desorption ionization time of flight (MALDI-TOF) experiments were performed on Bruker Autoflex MALDI-TOF instrument using laser power of 55-100%. The sample was dissolved in ultra-pure water (4 mg/mL) and the matrix was dissolved in acetonitrile:water mixture [50:50 (v/v)] at 10 mg/mL concentration. 10 µL of the dendrimer solution was mixed with 10 µL of the matrix solution to prepare the samples, out of which 3 µL of the solution was spotted on a MALDI plate.

#### Dynamic light scattering (DLS) and Zeta potential (ζ)

The size and the zeta potential distribution of the D-2PMPA were analysed using Zetasizer Nano ZS (Malvern Instrument Ltd. Worchester, U.K.) equipped with a 50 mW He-Ne laser (633 nm). The size measurements in triplicates were performed. The sample for size distribution measurement was prepared by dissolving the D-2PMPA in deionized water at a concentration of 0.5 mg/mL. The solution was filtered through 0.2 µm syringe filters (Pall Corporation, 0.2 µm HT Tuffryn membrane). The measurement was performed in a UV transparent disposable cuvette having dimensions as 12.5 × 12.5 × 45 mm (SARSTEDT). The zeta potential measurements were also performed in triplicates using a sample concentration of 0.2 mg/mL in 10 mM NaCl after the sample was filtered through 0.2 µm syringe filters.

### Primary glial cultures

We used primary mixed glial cultures to demonstrate cellular localization and efficacy of D-PMPA. Primary mixed glial cells were collected from neonatal rabbits (postnatal day 1) based on our previously established protocol 27. Neonatal rabbits were utilized to enhance yield and cell survival. Mixed glia were maintained in DMEM media containing 4.5 g/L glucose and 1.4 mM L-glutamine (Corning Cellgro, Manassas, VA USA) with 10% FBS and 1% pen/strep antibiotic at 37 °C and 5% CO_2_ atmosphere.

### Uptake studies in primary glial cultures

*In vitro* uptake of fluorescently labeled Cy5-D-2PMPA was assessed in primary mixed glial cultures as previously reported 27. Cells were plated into glass-bottom culture dishes and treated with 50 µg/mL Cy5-D-2PMPA in 5% serum medium. At particular time points (3, 6, 12, 18 and 24 hours), the cells were fixed with 2% paraformaldehyde for 15 minutes. The fixed cells were blocked with 5% normal goat serum for 4 hours followed by incubation with anti-Iba-1 antibody (1:300, Abcam, Cambridge, UK) for 12 hours at 4 °C to label microglial cells. Next the cells were washed with tris buffer with 0.1% triton X and were incubated with secondary antibody goat anti mouse Cy3 (1:500, Invitrogen, Waltham, MA) for Iba-1 and anti-GFAP (1:500, eBioscience, San Diego, CA, USA) overnight at 4 °C to stain astrocytes. The cells were washed twice with PBS for 5 minutes, stained with 4',6-diamidino-2-phenylindole (DAPI) (1:1000) (Invitrogen) for 15 minutes, and imaged under an LSM 710 confocal microscope (Carl Zeiss, Hertfordshire, UK) for identification of the dendrimers (Cy5-D-2PMPA) in microglia and astrocytes.

### Anti-inflammatory effect of D-2PMPA in LPS-treated primary glial cultures

Primary rabbit mixed glial cells cultured from neonate pups of both sexes were seeded onto 12-well plates. Rabbits were utilized due to increased cell yield versus mice, as dendrimers have been successfully employed in both species 13, 28, 29, along with rats 30, canines 31, and primates 32. After 48 hours, cells were activated with lipopolysaccharides (LPS; lot# 127M4130V, Sigma, St. Louis, MO USA) at 300 EU/mL for 6 hours. After 6 hours, the cells were treated with various concentrations of D-2PMPA (10-200 μg/mL) for 24 hours. For viability analyses, 5 mg/mL MTT solution was added for 4 hours and samples were analyzed at 540 nm. For rt-qPCR, cells were incubated in fresh media for 24 hours, followed by collection into Trizol (Invitrogen) and RNA extracted per manufacturer's instructions. RNA was then converted into cDNA for rt-qPCR analysis. Primer sequences:

- TGFβ: F: TGAGAGGTGGAGAGGAAATAGA, R: GGAACTGATCCCGTTGATGT;
- mGluR3: F: CGACAAGTCTCGCTACGATTAC, R: CACGTAGGTCCAGTTGAAGAAG;
- NR2A: F: CAAGGATCCCACGTCTACTTTC, R: AAGACGTGCCAGTCGTAATC;
- TNFα: F: TAGTAGCAAACCCGCAAGTG, R: CTGAAGAGAACCTGGGAGTAGA;
- iNOS: F: CAGGACCACACCCCCTCGGA, R: AGCCACATCCCGAGCCATGC;
- GAPDH: F: TGACGACATCAAGAAGGTGGTG, R: GAAGGTGGAGGAGTGGGTGTC.

For NAAG analysis, supernatants were collected. NAAG and NAA were individually spiked into HBSS to generate a standard curve from 0.1 to 100 nM. Fifty microliters of each sample or standard was added to an LC vial and spiked with 5 µL of 10 µM deuterated NAA (NAA-d3) as internal standard and vortexed. Samples were analyzed on an UltiMate 3000 UHPLC coupled to Q Exactive Focus orbitrap mass spectrometer (Thermo Fisher Scientific Inc., Waltham MA). Samples were separated on a Waters Atlantis dC18 (3 µm) 2.1 × 150 mm column. The mobile phase consisted of water + 0.1% formic acid (A), and acetonitrile + 0.1% formic acid (B). Separation was achieved at a flow rate of 0.4 mL/min using a gradient run, from 97.5/2.5 (A/B) to 5/95 (A/B) over 1.5 minutes. Quantification was performed in positive ion product-reaction monitoring mode with collision energy setting of 13 and 10 CE for NAAG and NAA respectively. Data were acquired and quantified with Xcalibur software.

### Mice

Female 7-week-old C57BL/6J mice were purchased from Jackson Laboratory (Bar Harbor, ME) and housed in the Miller Research Building Johns Hopkins animal facility. All protocols were approved by the Johns Hopkins Institutional Animal Care and Use Committee and cared for in compliance with the National Institutes of Health guide for the care and use of Laboratory animals (NIH Publications No. 8023, revised 1978).

### EAE immunizations and scoring

8-10-week-old mice were immunized for EAE as previously described 8 with minor modifications. Briefly, mice were administered murine MOG 35-55 (Johns Hopkins Peptide Synthesis Core Facility, Baltimore) in incomplete Freund's adjuvant (Sigma-Aldrich) supplemented with heat-killed mycobacterium tuberculosis (Sigma-Aldrich) via two subcutaneous flank injections. On days 0 and 2, mice received an intraperitoneal injection of 250 ng pertussis toxin (List Biological Laboratories). EAE disease scores were assigned by an observer blinded to the treatment as previously described 9 based on the following scale with 0.5 increments for intermediate scores: 0=normal, 1=limp tail, 2=wobbly gait, 3=dragging hind flank, 4=hind limb paralysis, 5=quadriplegia. Experiments were conducted in duplicate.

### D-2PMPA administration

To evaluate the effects of D-2PMPA treatment, vehicle (empty dendrimer) and D-2PMPA solutions in 0.9% sterile saline were prepared fresh every 2 weeks and stored at 4 ºC. Twice per week starting at 2 weeks post-immunization and continuing through sacrifice, mice (n=10) received intraperitoneal injections of vehicle or 20 mg/kg D-2PMPA (2-PMPA equivalent) at a volume of 10 μL/g body weight. Prior to treatment administration, solutions were brought to room temperature and vortexed.

### Glial uptake studies in EAE-immunized mice

To evaluate *in vivo* dendrimer uptake, a separate cohort of 3 mice were immunized for EAE, sacrificed, and brains were processed and stained for immunohistochemical analysis. Mice were injected intraperitoneally with 55 mg/kg Cy5-D-2PMPA conjugates on Day 14 post-immunization, and sacrificed 24 hours later via cardiac perfusion with ice cold PBS under anesthesia. Brains were dissected and post-fixed in 10% neutral buffered formalin for 48 hours at 4ºC. Brains were then moved through 15% then 30% sucrose solutions (Thermo Fisher Scientific, Waltham, MA) over 48 hours at 4 ºC, frozen in Tissue-Tek O.C.T. Compound (Electron Microscopy Sciences, Hatfield, PA) using 2-methylbutane and dry ice, then stored at -80 ºC until sectioning. Brain tissues were sectioned using a cryostat (Microm HM 505E, International Medical Equipment, MI, USA) at a thickness of 30 µm. Sections were permeabilized and blocked for 1 h at room temperature in 0.3% Triton X-100 (Sigma-Aldrich)/5% goat serum (Jackson Laboratories), then stained overnight at 4 °C with a primary antibody against microglia (Iba1; 1:500; Wako, Richmond VA) and astrocytes (GFAP; 1:500; Abcam). Sections were then stained with secondary antibodies for Iba-1 (goat anti-rabbit AlexaFluor 555; 1:1000; Invitrogen) and GFAP (goat anti-chicken AlexaFluor 488; 1:1000; Invitrogen) for 1 h at room temperature. The slides were then washed in 1X PBS 3 times for 5 minutes before being incubated with Hoechst 33342 (Thermo Fisher) for 5 minutes before being washed again and coverslipped with prolong diamond (Life Technologies). The slides were then imaged at 20× with a LSM800 confocal microscope (Zeiss). Images were processed using Zen Blue software (Zeiss).

### Cognition Studies

To evaluate cognition in EAE mice, the Barnes maze test was administered as previously described 8 approximately 5 weeks post-immunization. Briefly, mice were placed in the center of the maze (Maze Engineers, Glenview, IL) and trained to find a hidden target platform for 2 trials per day over 4 consecutive days. Primary latency, or the time lapsed between the start of the test and the mouse locating the target platform, and paths taken by the mice were automatically recorded. The researcher conducting cognition studies was blinded to the treatment groups. Dendrimer or D-2PMPA treatments were administered on the first and fourth day of testing, after all behavior testing was completed for the day.

Cohorts of normal Control C57BL/6 mice were separately tested in the same Barnes maze paradigm.

### Hippocampal CD11b+ cell isolations

Mice completing the Barnes maze cognitive test were delivered a terminal dose of dendrimer vehicle or D-2PMPA then sacrificed 24h later via cardiac perfusion with ice cold PBS. Hippocampal CD11b+ cells were isolated from a separate cohort of Control C57Bl/6 mice. Hippocampi, defined by landmarks and neuroanatomical nomenclature in the atlas of Franklin and Paxinos (anteroposterior: -0.95 - -4.03 mm, mediolateral: ±3.75 mm from bregma, dorsoventral: -1.75 - -5.1 mm from the dura) 33, were rapidly and bilaterally dissected on an ice-cold plate. CD11b^+^ cells were isolated from hippocampi as previously described 34. Briefly, brain tissue was minced in HBSS (Sigma-Aldrich, St. Louis, MO) and dissociated with neural tissue dissociation kits (MACS Militenyi Biotec, Auburn, CA). After passing through a 70 μm cell strainer, homogenates were centrifuged at 300 g for 10 minutes. Supernatants were removed, cell pellets were resuspended, and myelin was removed by Myelin Removal Beads II (MACS Militenyi Biotec). Myelin-removed cell pellets were resuspended and incubated with CD11b MicroBeads (MACS Militenyi Biotec) for 15 minutes, loaded on LS columns and separated on a quadroMACS magnet. Cells were flushed out from the LS columns, then washed and resuspended in sterile HBSS (Sigma-Aldrich). Viable cells were counted with a hemacytometer and 0.1% trypan blue staining. Each brain extraction yielded approximately 2.5×10^5^ viable CD11b^+^ cells. Samples were stored at -80 °C until GCPII activity assays were performed.

### GCPII activity assay

To determine the IC_50_ values of 2-PMPA, 2-PMPA-PEG-azide, D-2PMPA and the unconjugated generation 4 dendrimer (G4-OH), reactions were carried out in the presence or absence inhibitors, NAA-[^3^H]-G (30 nM, 48.6-49.6 Ci/mmol) and human recombinant GCPII enzyme (40 pM final) in Tris-HCl (pH 7.4, 40 mM) and 1 mM CoCl_2_. The reactions were carried out at 37 °C for 20 minutes and stopped with ice-cold sodium phosphate buffer containing 1mM EDTA (pH 7.4, 0.1 M, 50 µL). 90 µL aliquots from each terminated reaction was then transferred to 96-well spin columns containing AG1X8 ion-exchange resin and the plate centrifuged at 990 rpm for 5 minutes using a Beckman GS-6R centrifuge equipped with a PTS-2000 rotor. NAA-[^3^H]-G was bound to the resin and [^3^H]-G eluted in the flow through. To ensure complete elution of [^3^H]-G, columns were washed twice with formate (1 M, 90 µL). The flow through and the washes were collected and 200 µL aliquots transferred to a solid scintillator-coated 96-well plate (Packard) and dried to completion. The radioactivity corresponding to [^3^H]-G was determined with a scintillation counter (Topcount NXT, Packard, counting efficiency 80%). Subsequently, IC_50_ curves were generated from CPM results, using both Microsoft Office Excel 2016 and IDBS's XLfit 5.5.0.5 macro embedded within Excel.

To confirm target engagement, GCPII activity measurements were carried out on isolated CD11b+ cells based on previously published methods 35, 36. Glial cell pellets were suspended in 150 µl ice-cold Tris buffer (40 mM, pH 7.5) containing protease inhibitors (Roche, Complete Protease Inhibitor Cocktail, 1 tablet in 5 ml) and sonicated using Kontes' Micro Ultrasonic Cell Disrupter (three pulses of 10s duration on ice, 30s between pulses). The resulting homogenates were spun down (16,000 × g for 2 min at 4 °C) and the supernatants collected for both GCPII activity and total protein analysis. GCPII reaction was initiated after the addition of cobalt chloride (1 mM) and NAA-[^3^H]-G (40 nM) pre-warmed to 37 °C. The reactions were carried out in 50 µl reaction volumes in 96-well microplates for 2 h min at 37 °C. At the end of the reaction period, the assay was terminated, un-hydrolyzed NAA-[^3^H]-G and [^3^H]-G separated, and radioactivity corresponding to [^3^H]-G measured as detailed above. Finally, total protein measurements were determined as per manufacturer's instructions using BioRad's Detergent Compatible Protein Assay kit and data presented as fmol/mg/h.

### Statistical Analyses

Statistical analyses were completed using GraphPad Prism 6.0. One-way ANOVA with Tukey's multiple comparisons post-hoc test measured differences in PCR studies. Repeated measures two-way ANOVA with Sidak's multiple comparisons post-hoc test measured D-2PMPA treatment effects on EAE disease scores and Barnes maze cognitive performance. Unpaired t-tests determined statistical significance of GCPII activity in microglia. P values <0.05 were considered statistically significant. Statistical analyses assistance and consultation were provided by the Johns Hopkins Institute for Clinical and Translational Research Biostatistics Program.

## Results

### Synthesis and characterization of D-2PMPA conjugate

The D-2PMPA conjugate was prepared by the covalent attachment of 2-PMPA molecules on the surface of hydroxyl PAMAM dendrimers (D-OH) using copper (I) catalyzed alkyne-azide click chemistry (CuAAC). The conjugation of 2-PMPA on the surface of dendrimers was achieved in three steps: (i) the modification of the drug to attach a linker; (ii) partial modification of the surface of dendrimer with another linker to bring complementary functional group and finally, (iii) clicking the drug-linker to the dendrimer linker. The linkers were attached both to the dendrimer and the drug (a) to bring environment sensitive linkages for intracellular drug release, (b) to obtain complementary functional groups for conjugation, and (c) to reduce the steric hindrance for optimal drug loading.

**Figure 1:**
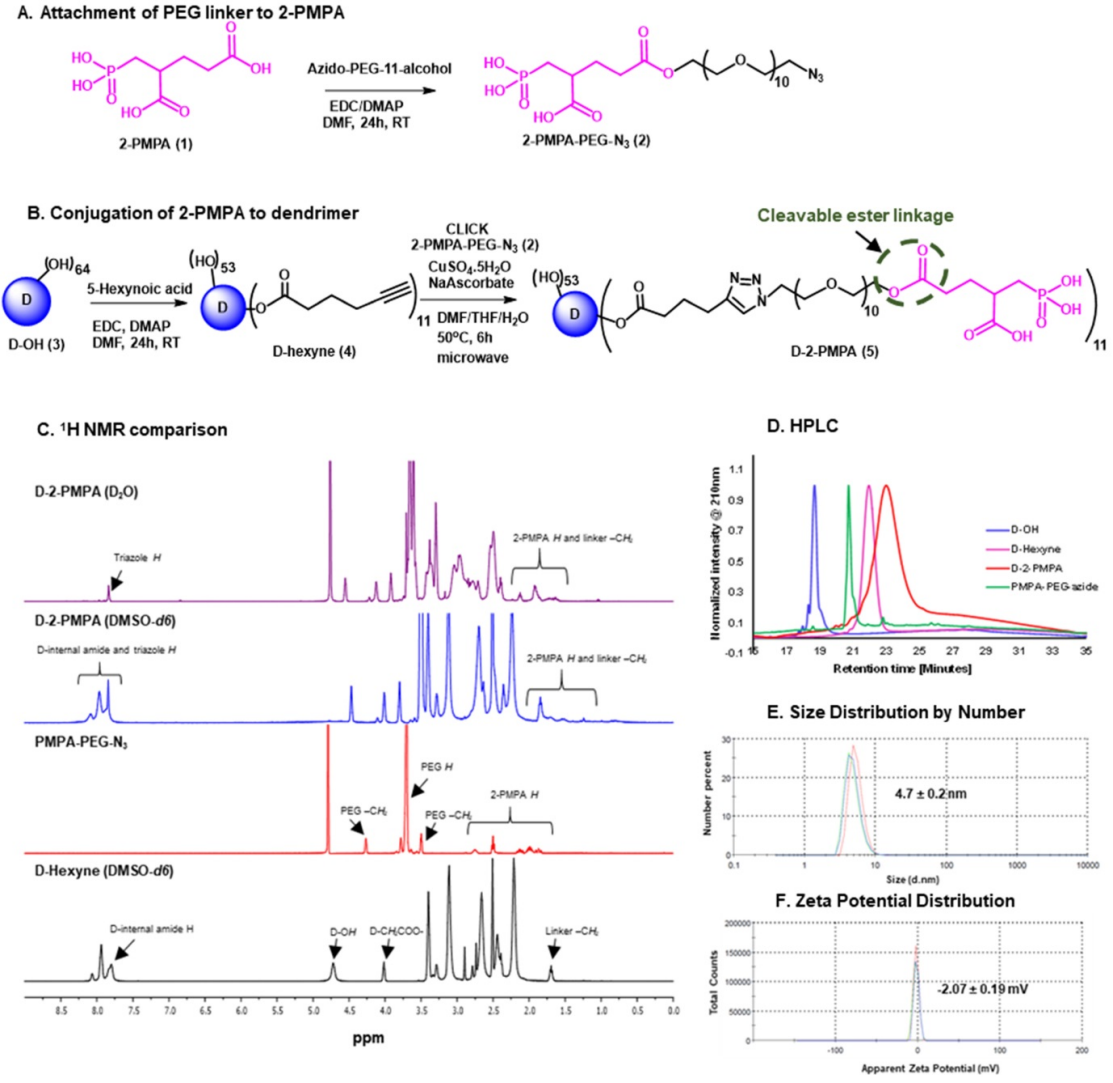
** Synthesis and characterization of D-2PMPA. A.** Synthetic protocol for the attachment of cleavable linker to 2-PMPA; **B.** The synthetic scheme for the conjugation of 2-PMPA on the surface of hydroxyl PAMAM dendrimer; **C.** Comparison of ^1^H NMR spectra for the intermediates and the final D-2PMPA conjugate; **D.** HPLC chromatogram showing distinct shifts at each reaction step; **E.** Size distribution by number of D-2PMPA conjugate, and **F.** Zeta potential distribution of D-2PMPA as analyzed by the dynamic light scattering.

The synthesis of D-2PMPA began with the modification of 2-PMPA to attach a short polyethylene glycol (PEG) linker with azide focal point to participate in the click reaction (**Figure 1A**). 2-PMPA (**1**) was reacted with azido-PEG-11-alcohol using EDC, DMAP as coupling agents. Compound **2** was purified by the reverse phase column chromatography. On the other hand, the surface of D-OH (**3**) was partially modified by the attachment of alkyne linker (**Figure 1B**). D-OH was reacted with hexynoic acid to bring approximately 11 alkyne moieties on the surface of dendrimer **4**. The dendrimer surface was only partially modified to maintain the inherent targeting potential of the hydroxyl dendrimer. The number of attached linkers was calculated by comparing the integration of dendrimer internal amide protons to the newly formed ester methylene protons in ^1^H NMR (**Figure 1C**). In the final step, D-hexyne (**4**) and 2-PMPA-PEG-azide (**2**) were clicked together via CuAAC click reaction using a catalytic amount of copper sulfate and sodium ascorbate to afford D-2PMPA (**5**). The confirmation of the click reaction was achieved by comparing proton NMR spectra of D-hexyne and PMPA-PEG-azide to D-2PMPA. The proton NMR spectra of D-2PMPA clearly showed the presence of 2-PMPA protons and shifts in the methylene protons adjacent to the triazole ring. The number of 2-PMPA molecules conjugated on dendrimer were analyzed by comparing 2-PMPA protons to dendrimer protons confirming the attachment of an average of 11 drug molecules with a loading of ~10% weight by weight. Due to the overlap with dendrimer internal protons, the appearance of triazole ring protons was not evident when NMR was taken in DMSO-*d6*. The ^1^H NMR spectra of D-2PMPA clearly showed the appearance of triazole H (δ 7.87 ppm) in the spectra obtained in D_2_O confirming the success of conjugation. D-2PMPA was further analyzed by HPLC which showed a clear shift in retention time for the conjugate (23.08 minutes) from the starting alkyne (22.0 minutes) and azide (20.74 minutes, **Figure 1D**). The HPLC purity of D-2PMPA was >99.5% (**Figure S5**). The size of D-2PMPA was 4.7 nm and zeta potential was nearly neutral (-2.07 mV) as analyzed by dynamic light scattering (**Figure 1E, F**).

### Synthesis and characterization of fluorescently-labeled Cy5-D-2PMPA conjugate

**Figure 2:**
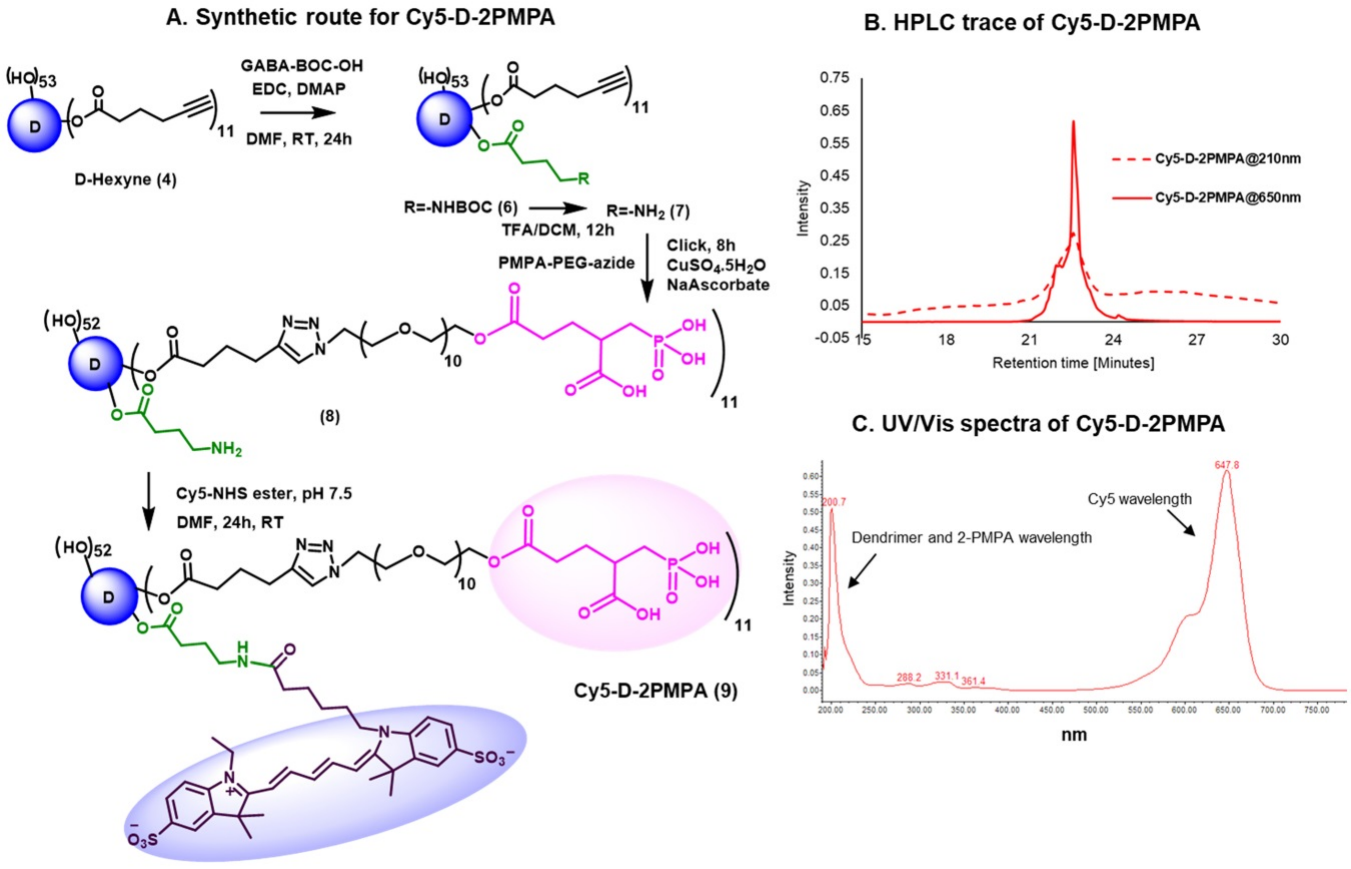
** Synthesis and characterization of Cy5-D-2PMPA. A.** Synthetic protocol for the synthesis of fluorescently-labeled dendrimer-2-PMPA conjugate (Cy5-D-2PMPA); **B.** HPLC chromatogram of Cy5-D-2PMPA at dendrimer absorption wavelength (210nm) and Cy5 absorption wavelength (650nm); and **C.** UV/Vis profile of Cy5-D-2PMPA showing absorption at both dendrimer and Cy5 wavelengths.

We further constructed fluorescently-labeled Cy5-D-2PMPA conjugates to perform *in vitro* and *in vivo* imaging to confirm that the attachment of 2-PMPA on the surface of dendrimer did not alter its targeting capabilities. The synthesis started with the modification of compound **4** to synthesize a multifunctional dendrimer with an amine group in addition to alkyne and hydroxyl groups (**Figure 2A**). This was achieved by the reaction of compound **4** with GABA-BOC-OH to obtain compound **6** with BOC protected amine which was then de-protected under mild acidic conditions using trifluoroacetic acid to yield multifunctional dendrimer **7**. The choice of the functional groups was made so that they did not interfere with each other during reactions. The alkynes in dendrimer **7** were further reacted with 2-PMPA-PEG-azide using classical click conditions as described earlier and the resulting compound **8** was obtained with approximately 11 PMPA molecules and an amine group. The dendrimer **8** was finally reacted with Cy5-NHS ester using activated acid amine coupling reaction to get Cy5-D-2PMPA. The structure of the final fluorescently-labeled conjugate was characterized using ^1^H NMR spectroscopy (**Figure S10**). The purity of Cy5-D-2PMPA was >99% as analyzed by HPLC (**Figure 2B and Figure S11**). The UV/Vis spectra of Cy5-D-2PMPA showed the absorption at both dendrimer and 2-PMPA absorption wavelength (200 nm) and Cy5 wavelength (650 nm) further confirming the formation of the conjugate (**Figure 2C**).

### *In vitro* evaluation of D-2PMPA

#### D-2PMPA inhibits human recombinant GCPII enzyme activity *in vitro*

**Figure 3:**
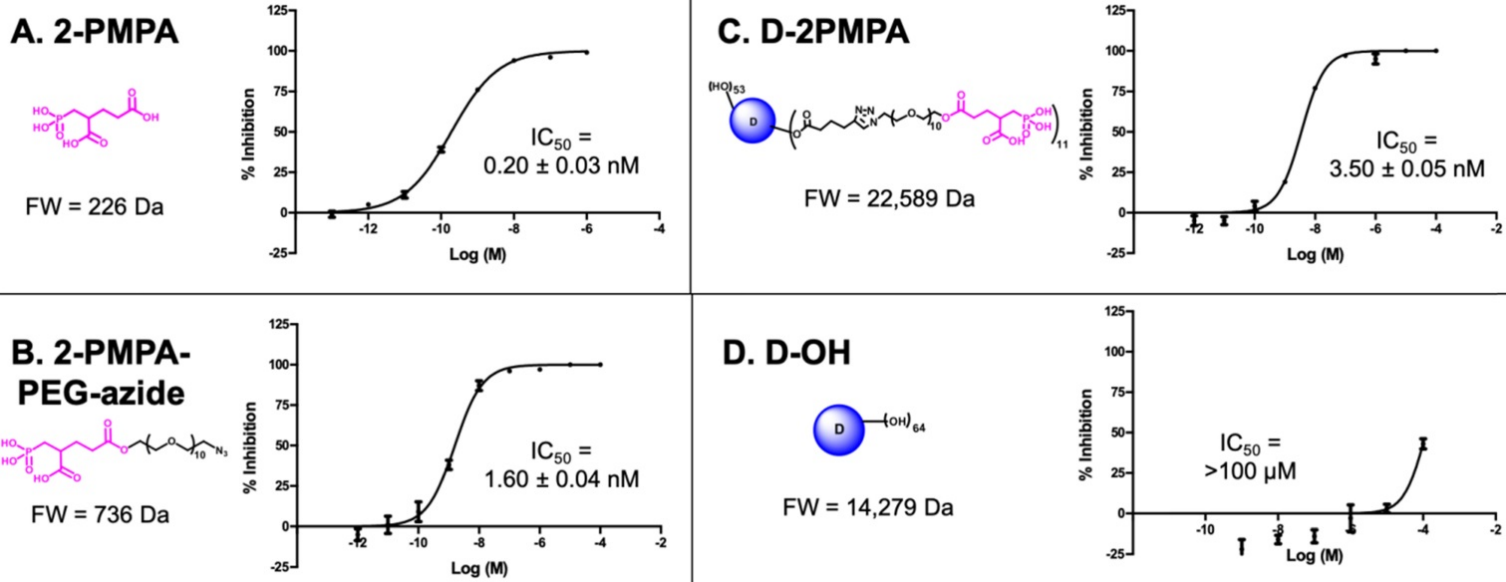
** The comparative IC_50_s of the free drug, drug linker and dendrimers determined using human recombinant GCPII**. **A.** 2-PMPA; **B.** 2-PMPA-PEG-azide; **C.** D-2PMPA; and **D.** D-OH.

To evaluate whether 2-PMPA conjugated to the dendrimer retained activity as an inhibitor of GCPII, inhibition was measured in the presence of D-2PMPA and compared with free 2-PMPA, 2-PMPA-PEG-azide, and D-OH (**Figure 3**). The IC_50_ of D-2PMPA was 3.50 ± 0.05 nM, an approximate 10-fold loss of potency relative to free 2-PMPA, 0.20 ± 0.03 nM. 2-PMPA conjugated to PEG-azide showed similar activity with an IC_50_ of 1.60nM ± 0.04 nM, while negative control D-OH displayed no inhibitory activity.

#### D-2PMPA localizes primarily to microglial cells in mixed glial cultures

**Figure 4:**
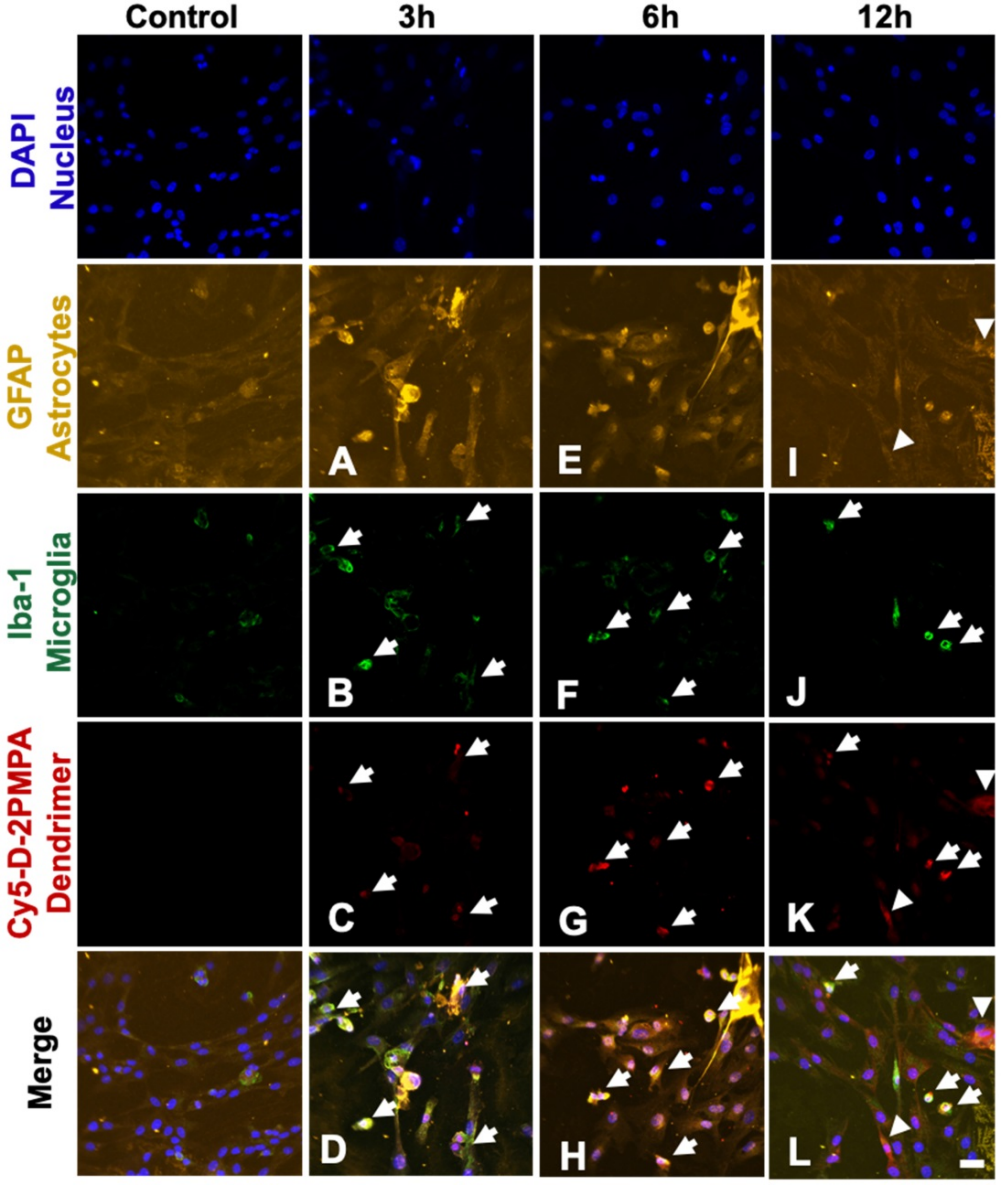
** Confocal images of glial cultures demonstrating Cy5-D-2PMPA uptake.** Microglia were stained using Iba-1 (green), astrocytes were stained using GFAP (orange), nuclei were stained using DAPI (blue), and the D-2PMPA conjugates were labelled with Cy5 (red). Cells were treated with 50 µg/mL of Cy5-D-2PMPA to evaluate cellular uptake. At 3 and 6 hours post treatment, Cy5-D-2PMPA was preferentially taken-up by microglial cells (**A-D** and **E-H**). Microglial uptake of Cy5-D-2PMPA continued at 12 hours post-treatment (**I-L**, white arrows). At 12 hours post treatment, astrocytes also demonstrated some signs of uptake (**I-L**, white arrowheads). Scale bar 20 µm.

We used mixed glial cultures containing both microglia and astrocytes to demonstrate the uptake kinetics of Cy5-D-2PMPA in both cell populations and determine if preferential uptake occurred in one cell type over another (**Figure 4**). At early time points (3 and 6 hours), Cy5-D-2PMPA was found predominantly co-localized with microglia (Iba-1+) cells (**Figure 4A-D** and **E-H**, white arrows). At 3 hours, about half of the Iba1+ microglial cells demonstrated dendrimer uptake (qualitatively), and at 6 hours, almost all the microglia population demonstrated dendrimer co-localization. This preferential uptake of Cy5-D-2PMPA by microglial population in mixed glial cells was similar to D-Cy5 (without conjugated PMPA) uptake as reported previously 27 suggesting that conjugating 2-PMPA does not alter the cellular uptake of dendrimers. Twelve hours post Cy5-D-2PMPA treatment, microglial uptake was still evident, with a few of the GFAP+ astrocytes also demonstrating Cy5-D-2PMPA uptake (**Figure 4I-L**, white arrowheads respectively).

#### D-2PMPA is anti-inflammatory in LPS-treated glial cultures

**Figure 5:**
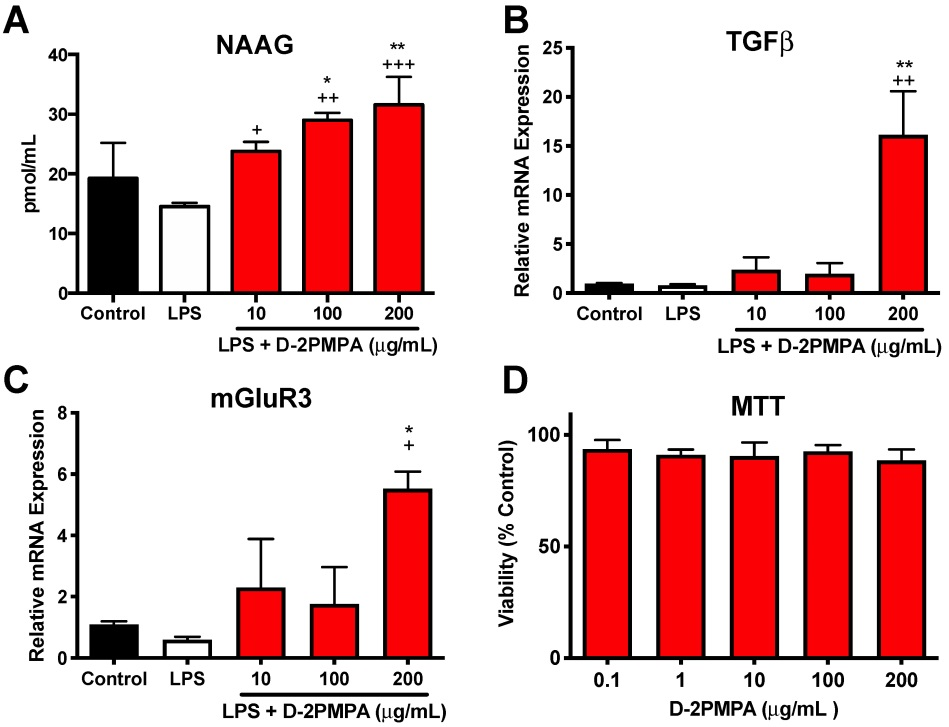
** Effect of D-2PMPA on LPS-treated glial cultures.** D-2PMPA treatment resulted in a dose-dependent upregulation of NAAG (**A**), TGFβ mRNA expression (**B**), and mGluR3 mRNA expression (**C**). D-2PMPA had no effect on cell viability as assessed by the MTT assay (**D**). Significantly different from control at *P<0.05, **P<0.01. Significantly different from LPS at ^+^P<0.05, ^++^P<0.01, ^+++^P<0.001.

Mixed glial cultures were used to evaluate the *in vitro* activity of D-2PMPA. Dendrimer delivery of 2-PMPA to LPS-treated glial cultures significantly increased NAAG, TGFβ, and mGluR3 (**Figure 5A-C,** P<0.05). A MTT assay revealed no toxicities at the concentration utilized (**Figure 5D**). D-2PMPA also caused a trend increase in NR2A, lowered levels of the oxidative stress marker iNOS, and significantly lowered levels of the pro-inflammatory cytokine TNFα (**Figure S12,** P<0.05).

### *In vivo* evaluation of D-2PMPA

#### D-2PMPA is taken up by microglia in EAE mice

**Figure 6:**
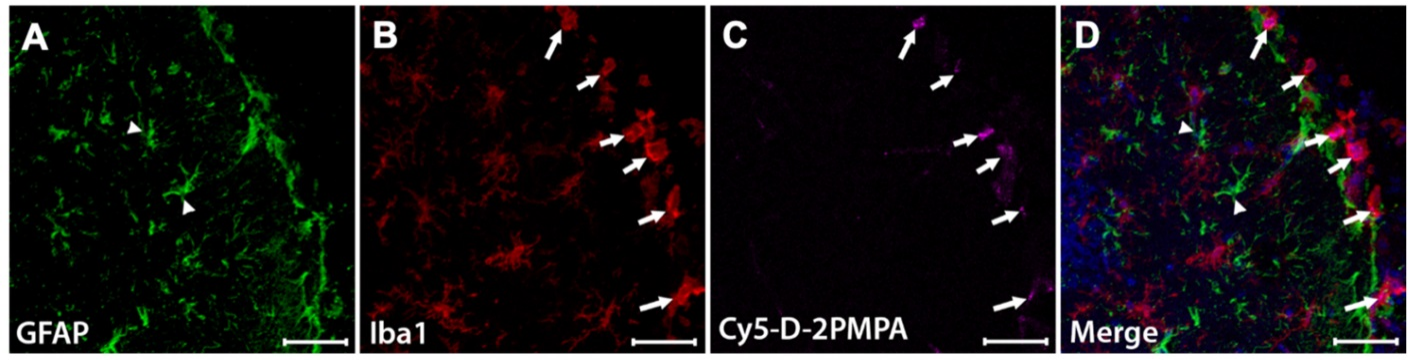
** Cy5-D-2PMPA is selectively taken up by activated microglia**. EAE mice were administered a single dose of Cy5-D-2PMPA at 14 days post-immunization and sacrificed 24 hours later. Representative brain images illustrate selective uptake Cy5-D-2PMPA (**C**, violet cells) in Iba1 positive activated microglia (**B**, red cells) along the edge of the dentate gyrus. Merged image of Iba1 and Cy5-D-2PMPA positive cells (**D**, pink cells). GFAP positive astrocytes (**A**, green cells) have no Cy5-D-2PMPA signal (**D,** arrowheads). Nuclei are stained blue in the merged image (**D**). Scale bars, 50 µm.

To evaluate D-2PMPA brain uptake following IP administration, EAE-immunized mice were administered a single IP Cy5-D-2PMPA dose on Day 14, sacrificed 24 hours later, and brains were imaged (**Figure 6**). A dose of 55 mg/kg was selected to improve visibility. Sections stained with Iba1 (red) confirmed perinuclear Cy5-D-2PMPA uptake in activated microglial cells. The highest concentrations of these positive cells were located in the molecular layer of the dentate gyrus near the third ventricle. No appreciable uptake was observed in astrocytes.

### D-2PMPA administration does not alter physical severity in EAE mice, but selectively improves cognitive function

**Figure 7:**
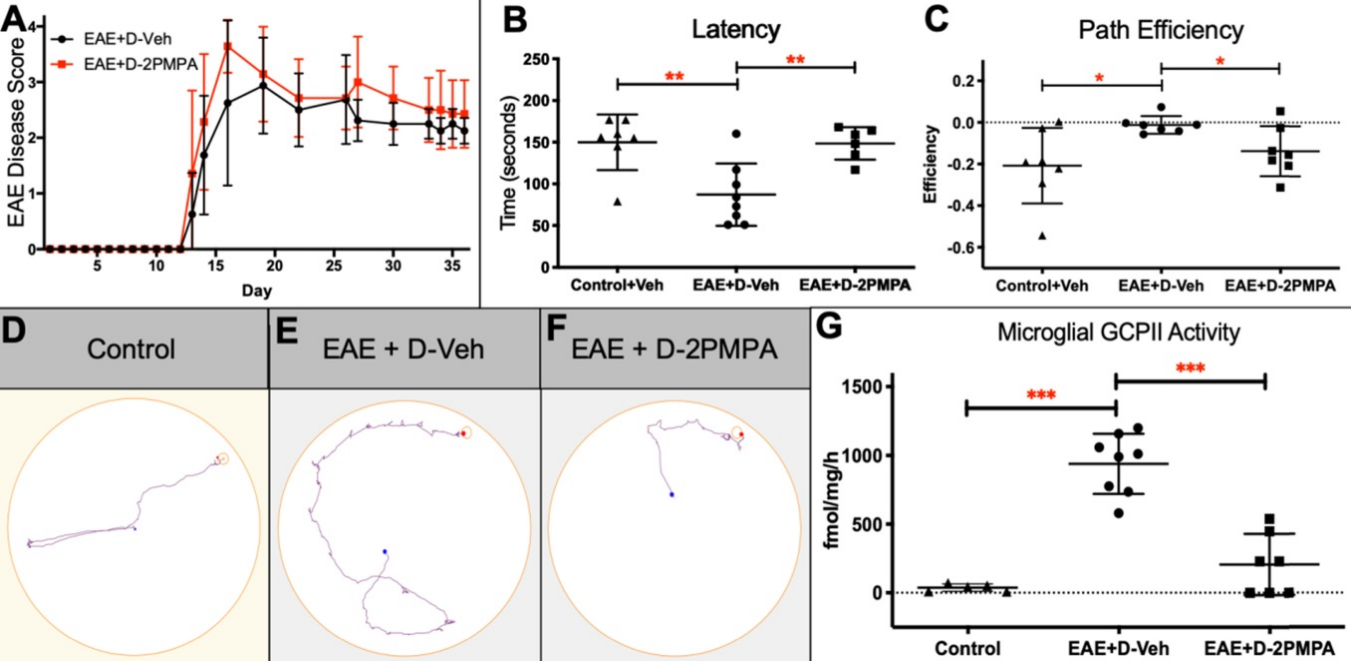
** D-2PMPA improves cognitive function and inhibits hippocampal GCPII activity in EAE mice.** Biweekly D-2PMPA (20mg/kg) treatment initiated at the onset of physical disease had no impact on EAE physical disease severity** (A)**. At 5 weeks post-immunization, EAE mice were tested in the Barnes maze. EAE mice exhibited impaired cognition function compared to non-EAE control mice that was reversed with D-2PMPA treatment as measured by the change between the first and last trial in latency to find the target **(B)** and path efficiency** (C)**. Representative track plot from the final Barnes maze trial illustrate the impaired performance in EAE mice versus controls that is improved with D-2PMPA treatment** (D-F)**. Experiments were performed in duplicate and data shown are mean ± SD of a representative experiment, n=7-8/group.** G.** Following Barnes maze testing, EAE mice received D-Veh or D-2PMPA and sacrificed 24 hours later and CD11b+ cells were isolated from hippocampi. GCPII enzymatic activity increased 24.7-fold in EAE mice treated with D-Veh versus Control mice. D-2PMPA treatment in EAE mice robustly attenuated the increased GCPII activity. Significantly different from EAE+D-Veh at P<0.001(***); Data are mean ± SD, n=5-8/group.

To determine the effects of GCPII inhibition on physical severity of EAE, mice were administered biweekly injections of either D-2PMPA (20 mg/kg) or empty dendrimer vehicle (D-Veh) from the time of physical EAE disease onset. A free 20 mg/kg 2-PMPA control group dosed biweekly was not included in the present studies because the drug is cleared from circulation within six hours, and previous dose response studies showed that a minimal dose of 100 mg/kg was required for positive behavioral effects and alterations in brain NAAG levels 9. Mice developed signs of EAE approximately 2 weeks post-immunization. Data from two independent experiments demonstrated that inhibition of GCPII via D-2PMPA treatment did not affect the severity or progression of EAE, as indicated by no detectable differences in EAE scores throughout the duration of the experiment (**Figure 7A**).

Following 3 weeks of biweekly vehicle or D-2PMPA treatment, cognitive function was evaluated using the Barnes maze test in the same cohorts of EAE mice monitored for disease score or in separate cohorts of Control mice. EAE mice treated with Vehicle demonstrated impaired learning and memory as evidenced by significantly reduced primary latency delta (first trial latency - final trial latency), while D-2PMPA treatment restored cognitive abilities to those of Control mice (**Figure 7B**, P<0.01). Path efficiency is the distance from the center starting point of the maze divided by the total distance traveled, with a score approaching 1 indicating the most efficient path to the target. The path efficiency delta (first trial efficiency - final trial efficiency) was significantly higher in EAE+D-Veh mice versus Controls, demonstrating impairments in EAE mice, while path efficiency was lower in D-2PMPA mice, demonstrating more proficient learning versus EAE+D-Veh mice** (Figure 7C**, P<0.05). Paths in the maze were recorded and automatically tracked, and representative track plots from the final trial of Barnes maze testing show restored cognitive function in EAE mice due to D-2PMPA treatment (**Figure 7D-F**).

### D-2PMPA inhibits GCPII activity in hippocampal CD11b+ cells from EAE mice

To confirm a disease-mediated upregulation in GCPII activity, along with target engagement following D-2PMPA treatment, GCPII enzymatic activity levels were measured in microglial-enriched cells of EAE mice that completed Barnes maze cognitive testing and a separate cohort of Control mice. While GCPII activity was low in hippocampal CD11b+ cells from Control mice, it was significantly upregulated in EAE-immunized mice (**Figure 7G**, 24.7-fold increase; P<0.001). D-2PMPA treatment led to a >75% reduction in GCPII activity levels in EAE mice when compared to EAE mice treated with vehicle dendrimer (P<0.001).

## Discussion

Here we synthesized a GCPII inhibitor-dendrimer conjugate by attaching 2-PMPA to the surface of hydroxyl PAMAM dendrimers and evaluated its ability to deliver 2-PMPA to activated glia and modulate cognitive performance in a murine EAE model of MS. 2-PMPA contains a dicarboxylic acid and a polar phosphonate, making it highly hydrophilic, resulting in low membrane permeability, negligible oral bioavailability (*F*<2%), and very limited brain penetration (AUC_brain_/AUC_plasma_<0.02) 37. In preclinical models of neurological diseases where 2-PMPA has shown therapeutic potential, it is efficacious only after very high systemic doses (i.p.) or direct brain injection, and is therefore not optimal for chronic dosing in patients 8, 9. To circumvent these issues, 2-PMPA was covalently conjugated to the surface of hydroxyl PAMAM dendrimers using an efficient, robust, and atom economical CuAAC approach 38, 39. Specific uptake of D-2PMPA into activated microglia was confirmed in glial cultures, and administration of D-2PMPA showed robust and dose-dependent anti-inflammatory activity. Systemically-administered D-2-PMPA greatly facilitated selective delivery to activated glial cells in the brains of EAE mice, where there was a 24.7-fold upregulation in GCPII activity in EAE mice compared to normal control mice. Employing a twice weekly dosing regimen initiated at the time of physical disease onset in order to mimic a clinical treatment paradigm, D-2PMPA significantly improved the cognitive impairment in EAE mice as assessed by Barnes maze performance and significantly lowered glial GCPII activity levels. Of note, the efficacious dose of D-2PMPA represents a 17.5-fold reduction compared to the dose of free 2-PMPA required to achieve a similar behavioral outcome 8.

Hydroxyl-terminated PAMAM dendrimers have a remarkable ability to deliver drugs to activated brain microglia. While hydroxyl PAMAM dendrimers do not cross an intact BBB, the impaired BBB in MS and other neurological diseases allows for brain penetration and subsequent dendrimer uptake into activated glial cells 15, 16. PAMAM dendrimers are nontoxic at doses up to 500 mg/kg in preclinical models, 25x higher than the therapeutic dose utilized in the present study, and are cleared intact through the kidney within hours if not taken up by activated cells 17, 40. These features make dendrimer delivery of small molecule therapeutics an extremely attractive drug delivery strategy for neurological diseases with glial activation and BBB disruption. In fact, hydroxyl PAMAM dendrimers have been extensively studied in multiple species, including primates, and numerous preclinical disease models, showing significant alleviation of neuroinflammation, oxidative stress, and neurologic injury 13, 17, 22, 32, 41. Positive preclinical findings led to clinical trial testing the safety and tolerability of dendrimer administration in humans (NCT03500627) and the initiation of a clinical trial to test the efficacy of dendrimer-conjugated N-acetyl-cysteine for COVID-19 (NCT04458298).

The present studies are focused on utilization of D-2PMPA for the treatment of MS-related cognitive impairment. Synthesis of fluorescently labeled Cy5-D-2PMPA allowed for visualization of dendrimer uptake in both cultured glial cells and in EAE brains. Similar to previously published reports, we observed very limited uptake of dendrimer conjugates into astrocytes as compared with microglia in culture studies, and no uptake into astrocytes in EAE brains 27, 42. To our knowledge, only one other study to date has tested a dendrimer drug delivery system in the EAE mouse model of MS, with positive anti-inflammatory results resulting from treatment with an amino-bis(methylene phosphonate)-capped dendrimer 43. Dendrimers have also proven efficacious in targeting activated glia in other models of neurological disease, including inflammatory preterm birth injury 20, cerebral palsy 13, and Rett Syndrome 14. Because glial cells are implicated in the pathogenesis of many neurological diseases and preclinical models, including EAE and MS 44, dendrimer drug delivery has the potential to assist in the generation of new targeted therapies. Furthermore, dendrimers may aide in the clinical translation of drugs with poor pharmacokinetic profiles that prevent CNS delivery, such as GCPII inhibitors, or drugs with peripherally mediated toxic side effects. Dendrimer-mediated drug delivery is particularly attractive for targeting GCPII. Although the enzyme is known to be expressed on the extracellular surface of glia, it can also be endocytosed by clathrin-based machinery 45, 46 or localized intracellularly following endoproteolytic cleavage 47, thereby allowing for drug delivery that is proximate to the desired target.

Multiple independent studies have demonstrated associations between brain NAAG levels and cognitive function both in human subjects and preclinical models. Reductions in brain NAAG levels have been observed in individuals with Alzheimer's and Huntington's diseases 48, 49, and brain NAAG levels were shown to correlate to cognitive function in patients with schizophrenia 50 and MS 8. Inhibition of GCPII activity has demonstrated benefit in a host of preclinical studies, including models of schizophrenia, Alzheimer's disease, ethanol intoxication, and pain 51, 52, in addition to MS 8, 9. A recent study provided more mechanistic evidence for a role of GCPII and NAAG in cognitive function 53. A missense mutation in the FOLH1 gene that codes for GCPII (rs202676) was found to be associated with both increased GCPII expression and decreased brain NAAG levels in both patients with psychosis and unaffected healthy individuals. Remarkably, this mutation resulted in a dramatic reduction in IQ score (p=0.00037). In addition, the reduced brain NAAG levels were directly associated with worse cognitive performance. Furthermore, patients with the missense mutation had higher levels of cortical activity during tasks of working memory, indicating that their brains had to work harder to complete the tasks 53. This work underscores the importance of GCPII and NAAG in regulating cognitive function in both healthy individuals and those with neurological diseases.

The release of NAAG and subsequent cleavage of the neuropeptide by GCPII is elevated under conditions of high synaptic activity 5, such as neuropathological injury in MS. NAAG can be synthesized in neurons or glial cells 54-56 and is an agonist at mGluR3, which plays a critical role in cognitive processes. mGluR3 knockout mice exhibit pronounced cognitive impairment versus wild type counterparts 57 and variations in the mGluR3 gene GRM3 are associated with poor cognitive performance and worsened cortical activity in human subjects 58. Multiple laboratories have confirmed that the positive effects of GCPII inhibition can be reversed following administration of mGluR3 antagonists 7, 59, 60. Activation of mGluR3 enhances the release of neurotrophic factors such as TGFβ 61, 62, which is required for GCPII to afford neuroprotection 63. In the present study, D-2PMPA treatment increased microglial release of NAAG, mGluR3, and TGFβ in culture, and we hypothesize that that the observed behavioral improvements in the present study are also due to NAAG-mediated stimulation of the mGluR3 pathway. In support of this hypothesis, it was recently reported that mGluR3 enhances synaptic strength in primate dorsolateral prefrontal cortex via cAMP-PKA-K+ signaling regulation, and that NAAG-mediated mGluR3 stimulation enhances delayed cell firing during working memory tasks 64, 65. In line with our findings, a recent study reported a downregulation in microglia mGluR3 gene expression in a rat model of perinatal brain injury 66. Similarly, mGluR3 gene expression was upregulated in individuals with MS who responded positively to interferon beta therapy 67. There are, however, some conflicting studies that report an upregulation in mGluR3 message in response to inflammatory conditions 68, 69, including MS 70. Discrepancies between these results and ours are could be due to different methodologies, including the use of nonspecific group II mGlurR antibodies 70, and model systems, along with looking at acute, subacute, or chronic stages of inflammation. Our data reflect a single time point following 30h of LPS exposure and 24h of D-2PMPA exposure, and it is likely that changes in exposure time would alter results. Future studies will examine time course changes in mGluR3 expression in culture, along with gene expression at different stages of EAE.

EAE is a highly inflammatory disease, and previous studies have shown that both TNFα and iNOS can contribute to EAE disease progression 71, 72. Along with an upregulation of anti-inflammatory markers we also observed a D-2PMPA-mediated downregulation in pro-inflammatory markers, so it was somewhat surprising to not observe changes in physical EAE severity due to D-2PMPA treatment. These findings, however, are in line with our previous observations that free 2-PMPA treatment had no effect on EAE disease score 8, 9. In addition to contributing to EAE disease progression, elevations in TNFα can cause synaptic instability and have been linked to cognitive dysfunction in EAE 73. Additionally, reductions in both TNFα and iNOS levels are associated with improved cognition in rats 74, so it is possible that targeted brain microglial uptake drove the behavioral results in the present study. Others, however, have reported a positive effect of GCPII inhibition on physical signs of disease, and we acknowledge that it is possible that with a modified treatment paradigm (i.e. earlier or more frequent dosing, higher *in vivo* doses, or combination therapy) EAE physical severity would be impacted. Future studies will explore this idea.

Female mice were selected in the present study in an attempt to both represent the disproportionately higher number of females impacted by MS 75, and to achieve a more reliable EAE disease phenotype. Male mice are more susceptible to early life handling-induced changes in disease susceptibility 76, and reproducible and consistent disease severity is critical in behavioral studies. However, it is necessary to recognize this limitation, and future studies will measure therapeutic effects of D-2PMPA in male EAE mice.

As treatments that target MS-related disability improve and lifespan is prolonged, it is reasonable to predict that comorbidities such as cognitive impairment will manifest to a greater degree. In turn, the negative effects of cognitive impairment on social well-being and financial productivity are therefore likely to magnify. While small to moderate effects have been observed due to computerized cognitive training 77, no drug therapies are currently designed and approved to treat cognitive impairment in MS. Inhibition of GCPII via targeted dendrimer drug delivery, therefore, has the potential to serve as the first therapeutic strategy to target MS-related cognitive impairment.

## Conclusion

Using hydroxyl PAMAM dendrimers, we developed the potent brain-penetrant, microglial-targeted GCPII inhibitor D-2PMPA. Selective uptake of D-2PMPA into microglia was confirmed both in glial cultures and EAE-immunized mice using Cy5-labelled-D-2PMPA. In LPS-treated glial cultures, D-2PMPA caused significant elevations in NAAG, TGFβ, and metabotropic glutamate receptor 3 (mGluR3). When evaluated in EAE mice, systemically administered D-2PMPA robustly inhibited the elevated GCPII enzymatic activity in CD11b+ brain cells and significantly improved cognitive function as assessed by Barnes maze performance at doses 17.5-fold lower compared to free 2-PMPA. Taken together, these data demonstrate the utility of hydroxyl dendrimers to provide targeted microglial delivery and support further development of D-2PMPA to attenuate elevated microglial GCPII activity and treat cognitive impairment in MS.

## Supplementary Material

Supplementary figures.

## Abbreviations

2-PMPA: 2-(phosphonomethyl)-pentanedioic acid
BBB: blood brain barrier
CNS: central nervous system
CuAAC: copper catalyzed alkyne-azide click chemistry
D-2PMPA: dendrimer-conjugated 2-PMPA
D-OH: hydroxyl PAMAM dendrimers
EAE: experimental autoimmune encephalomyelitis
GCPII: glutamate carboxypeptidase II
mgluR3: metabotropic glutamate receptor 3
MS: multiple sclerosis
NAA: N-acetylaspartate
NAAG: N-acetylaspartylglutamate
PAMAM: polyamidoamine
PEG: polyethylene glycol
