# Supplementary material for: Dendrimer-2PMPA selectively blocks upregulated microglial GCPII activity and improves cognition in a mouse model of multiple sclerosis
Source: Nanotheranostics. 2022 Jan 1;6(2):126–42. doi: 10.7150/ntno.63158 (PMC8671953; doi:10.7150/ntno.63158)
Supplement: Supplementary file 1 — Supplementary figures. [file ntnov06p0126s1.pdf]

Supplementary Figures

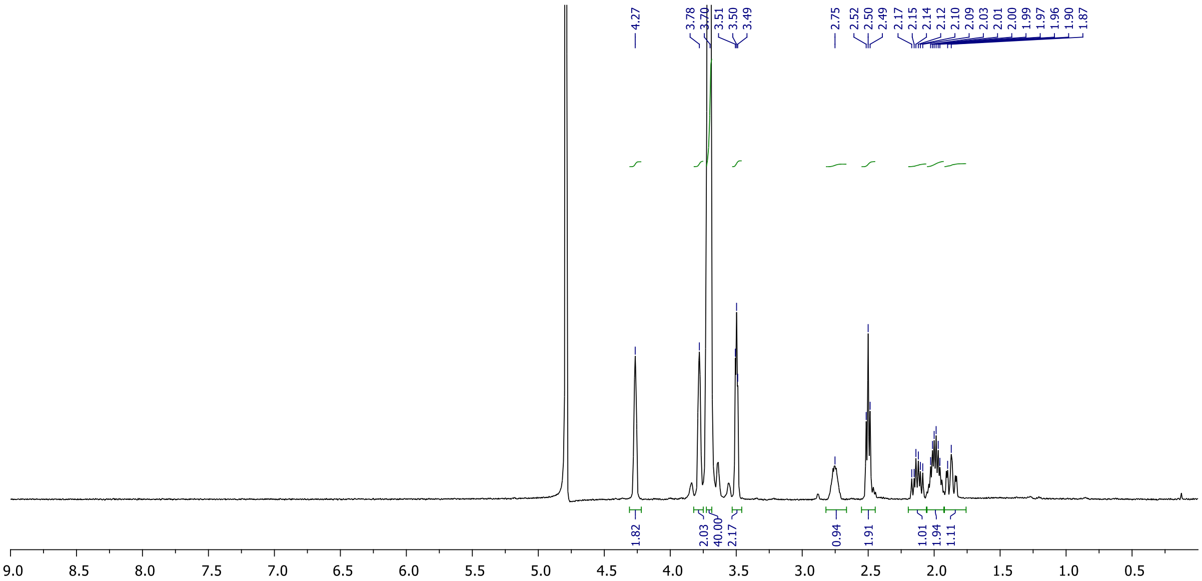

Supplementary Figure 1. ^{1}H NMR of 2PMPA-PEG-N_3 (compound **2)** in D_2O

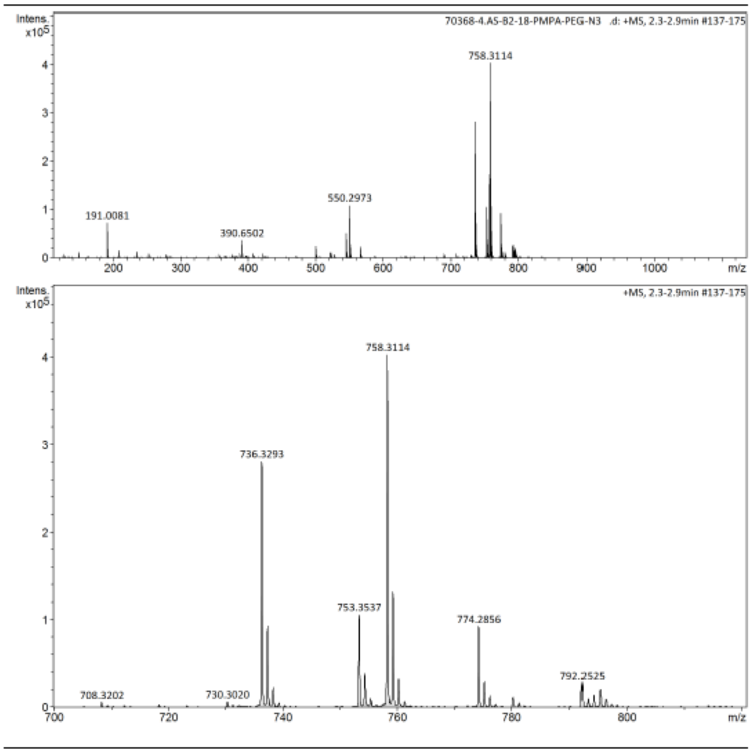

Supplementary Figure 2. ESI-MS spectra of 2PMPA-PEG-N_3 (compound **2)**

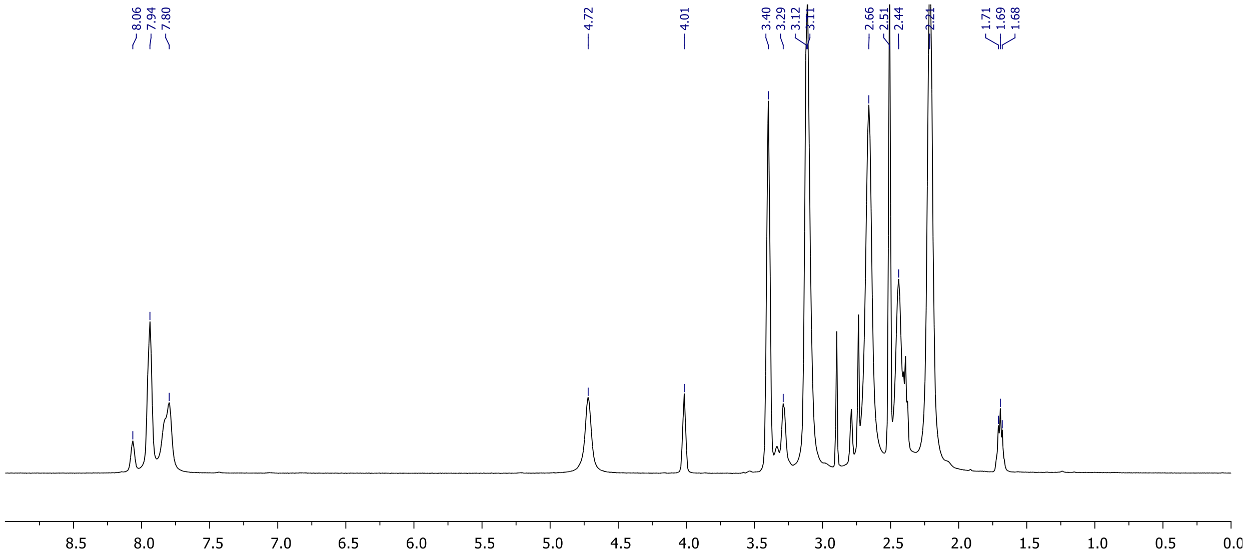

Supplementary Figure 3. ^{1}H NMR of D-hexyne (compound **4)** in DMSO-*d6*

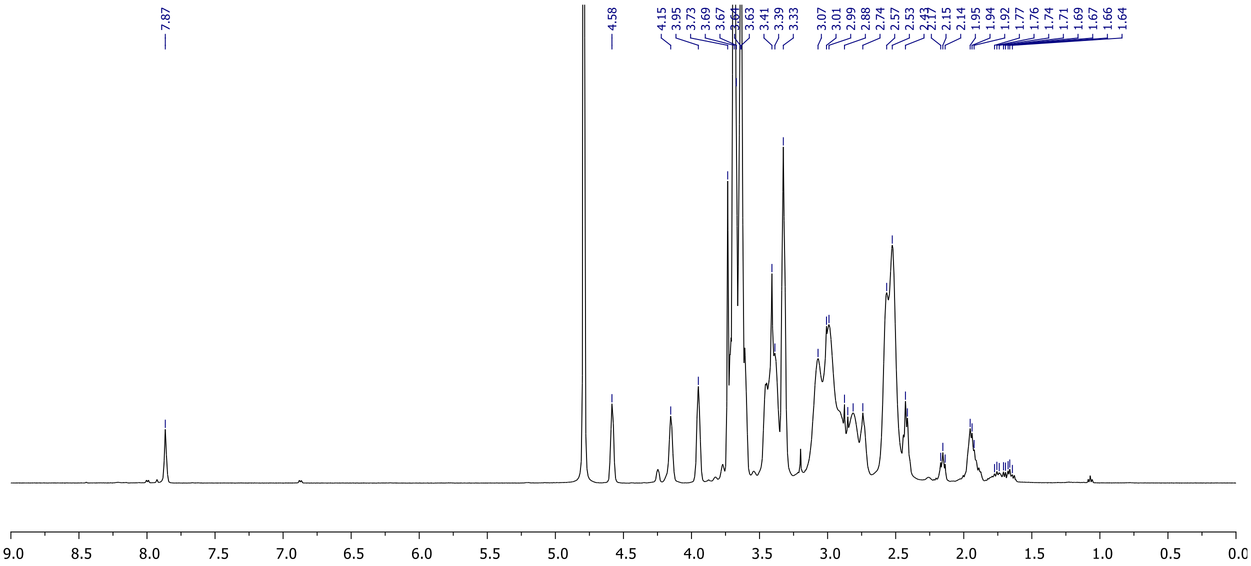

Supplementary Figure 4. ^{1}H NMR of D-2PMPA (compound **5)** in D_2O

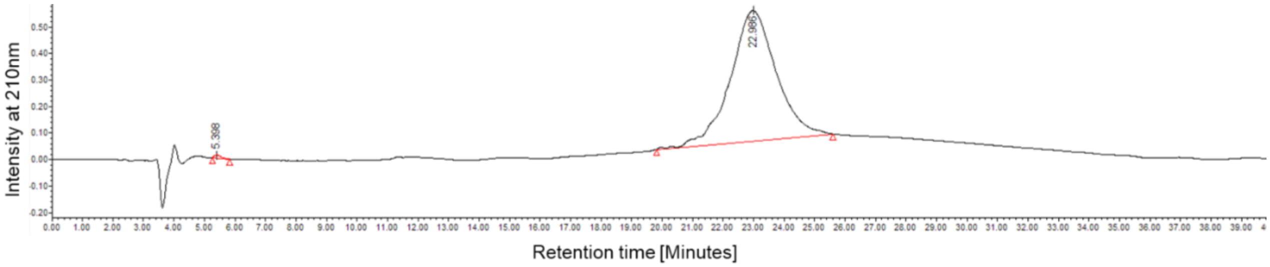

Retention Time (min)	Area (µV*sec)	% Area	Height (µV)
5.398	159949	0.31	11287
22.986	51298359	99.69	493039

Supplementary Figure 5. HPLC trace of D-2PMPA (compound **5)** at 210nm showing a purity >99%

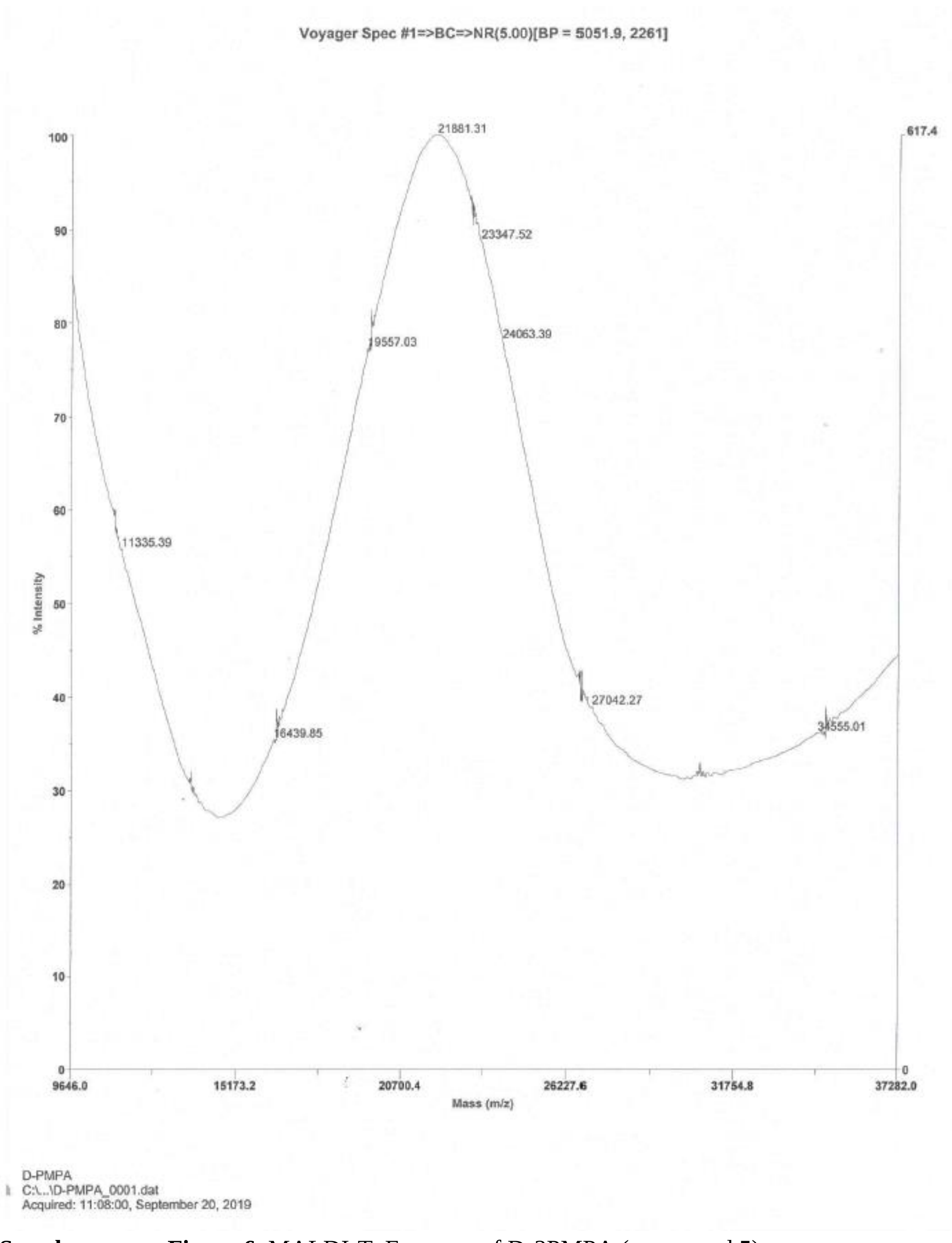

Supplementary Figure 6. MALDI-ToF spectra of D-2PMPA (compound **5**)

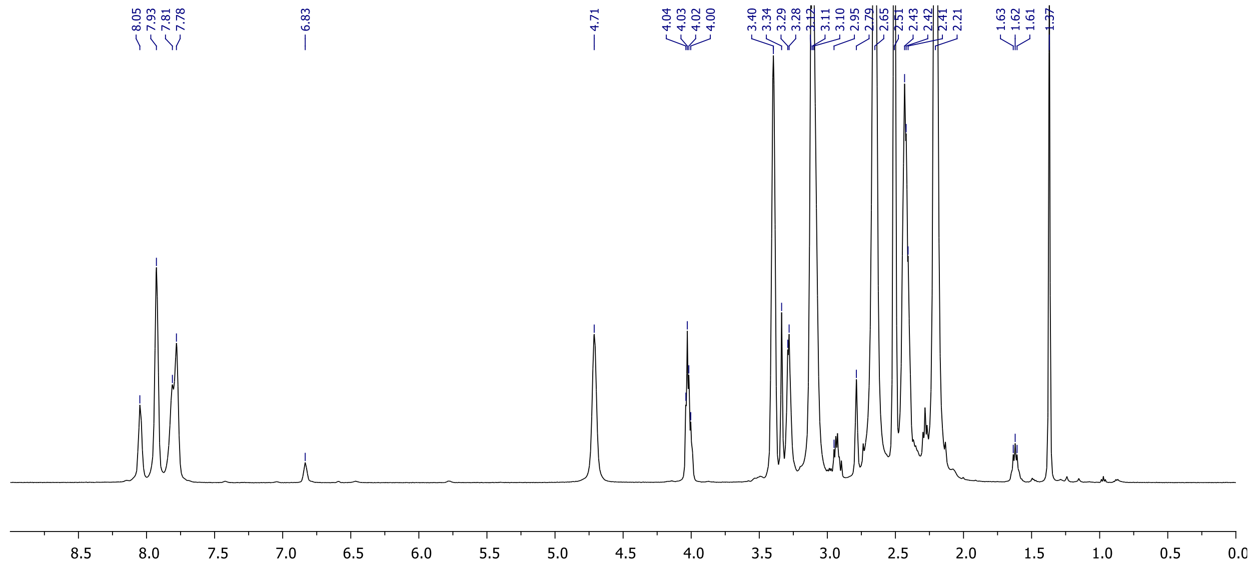

Supplementary Figure 7. ^{1}H NMR of compound **6** in DMSO-*d6*

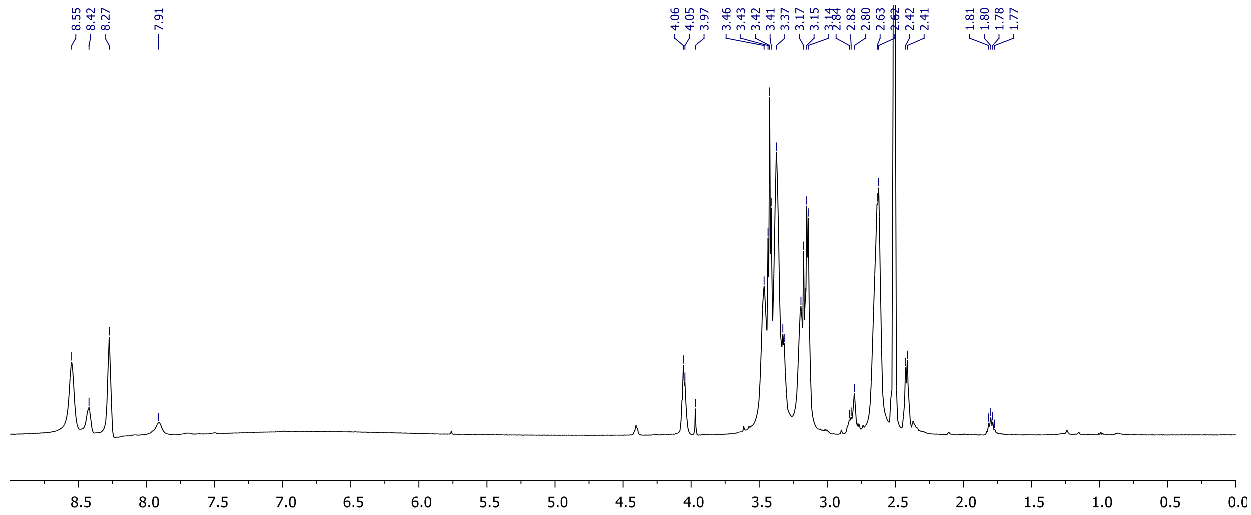

Supplementary Figure 8. ^{1}H NMR of compound **7** in DMSO-*d6*

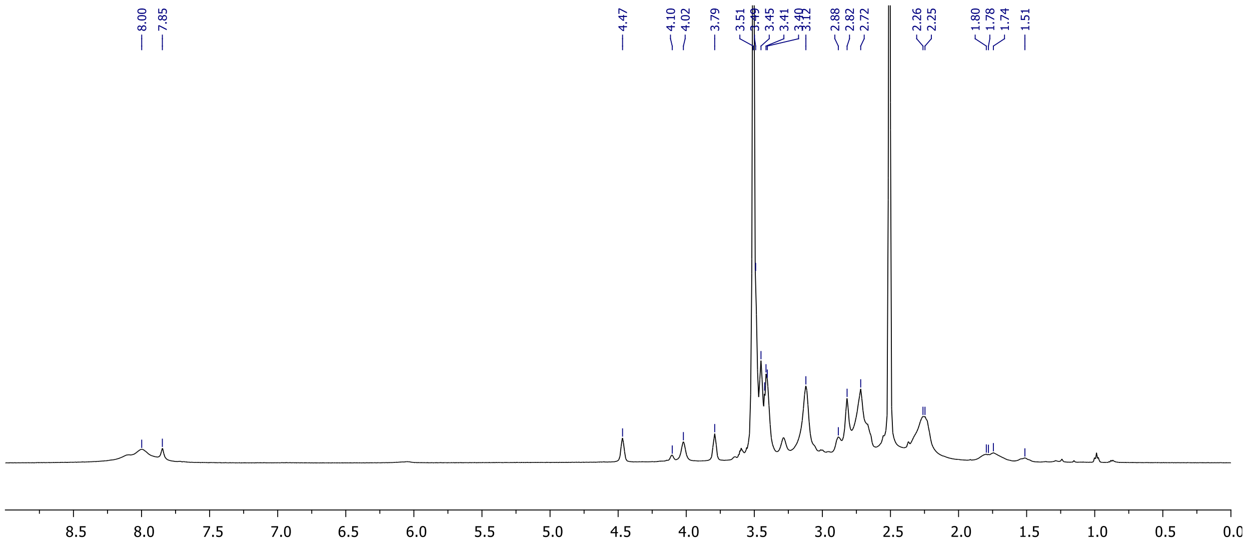

Supplementary Figure 9. ^{1}H NMR of compound **8** in DMSO-*d6*

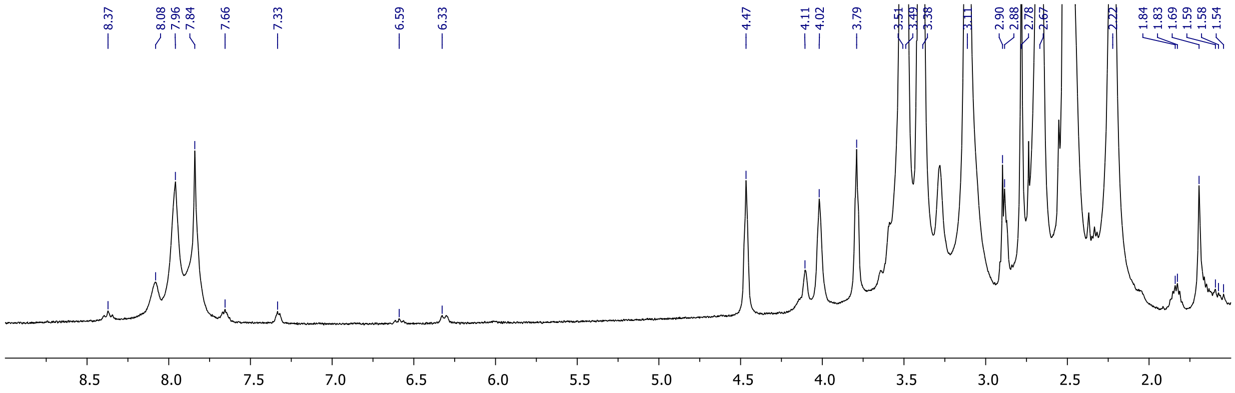

Supplementary Figure 10. ^{1}H NMR of Cy5-D-2PMPA (compound **9)** in DMSO-*d6*

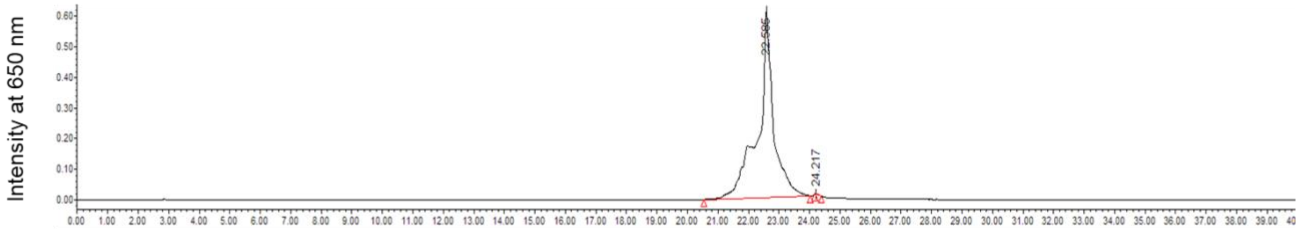

	Name	Retention Time (min)	Area (µV*sec)	% Area	Height (µV)
1		22.585	21353518	99.97	609742
2		24.217	5639	0.03	1441

Supplementary Figure 11. HPLC chromatogram of Cy5-D-2PMPA (compound **9)** showing greater than 99% purity

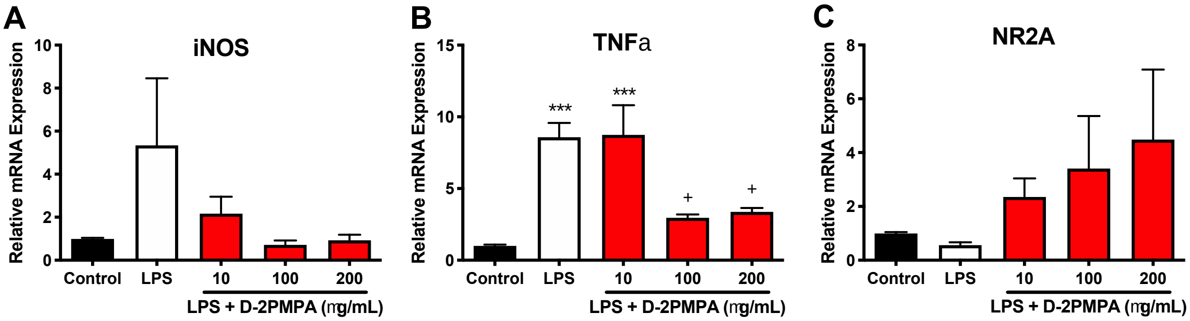

Supplementary Figure 12. Anti-inflammatory effect of D-2PMPA on LPS-treated glial cultures. D-2PMPA downregulates pro-inflammatory markers **A.** iNOS and **B.** TNFα and causes a trend increase in **C.** NR2A. Significantly different from control at P<0.001(***). Significantly different from LPS at P<0.05(+).
